# Mechanisms of self-other representations and vicarious experiences of touch in mirror-touch synesthesia

**DOI:** 10.3389/fnhum.2013.00112

**Published:** 2013-04-03

**Authors:** Michael J. Banissy, Jamie Ward

**Affiliations:** ^1^Department of Psychology, Goldsmiths, University of LondonLondon, UK; ^2^Institute of Cognitive Neuroscience, University College LondonLondon, UK; ^3^School of Psychology, University of SussexBrighton, UK

In recent years several studies have documented a near-universal tendency to vicariously represent the actions and sensations of others (e.g., see Keysers and Gazzola, 2009 for review). For example, observing another person experiencing pain activates neural regions involved in experiencing pain (e.g., Singer et al., 2004; Avenanti et al., 2005) or observing somebody being touched recruits regions of the somatosensory cortex involved in experiencing touch (e.g., Keysers et al., 2004, 2010; Ebisch et al., 2008; Schaefer et al., 2012). For most of us, these vicarious representations are implicit and do not lead to overt sensations of the observed events (e.g., we do not feel pain when observing pain to others). There are, however, a small number of individuals who do experience overt somatic sensations when observing others' tactile experiences (Ward et al., 2008; Osborn and Derbyshire, 2010; Fitzgibbon et al., 2012; Banissy, 2013). For example, in mirror-touch synesthesia observing touch or pain to others evokes a conscious tactile sensation on the synesthetes' own body (Banissy and Ward, 2007; Holle et al., 2011). This opinion piece seeks to discuss potential neural mechanisms that contribute to the developmental form of mirror-touch synesthesia (for descriptions of acquired forms of mirror-touch/pain synesthesia see Fitzgibbon et al., 2012; Goller et al., 2013), and the important role that self-other representations may have on vicarious experiences of touch in mirror-touch synesthesia.

Approximately 1.6% of individuals experience developmental mirror-touch synesthesia and there are at least two spatial subtypes (Banissy et al., 2009; also see White and Aimola Davies, 2012). In the more common subtype, the synesthetic experience is evoked as though looking in a mirror (i.e., observing touch to the left side of the face evokes tactile sensations on the right side of the synesthete's face). In the less common, anatomical subtype, the synesthetic experience is mapped anatomically (i.e., observing touch the left side of the face evokes tactile sensations on the left side of the synesthete's face)^1^. For each subtype, their experiences are reported to be automatic, enduring, and present since childhood (Banissy and Ward, 2007; Holle et al., 2011).

While several studies have examined cognitive and perceptual characteristics of mirror-touch synesthesia (e.g., Banissy and Ward, 2007; Banissy et al., 2009, 2011; Holle et al., 2011; White and Aimola Davies, 2012; Aimola-Davies and White, 2013), there has been relatively less research that delineates the neural mechanisms that contribute to developmental mirror-touch. One common suggestion is that developmental mirror-touch synesthesia may be a function of atypical cortical excitability within neural regions supporting normal somatosensory mirroring. That is, brain regions that are generally recruited when observing touch to others are over excitable in mirror-touch synesthesia leading to observed touch evoking overt tactile sensations. For example, Blakemore and colleagues (2005) reported the first case of developmental mirror-touch synesthesia in a functional neuroimaging study where they compared neural activity in a single mirror-touch synesthete (“C”) to a group of control participants. Using fMRI Blakemore and colleagues investigated the neural systems underlying C's synesthetic experience by contrasting brain activity when watching videos of humans relative to objects being touched (the latter did not evoke synesthesia) in “C” and in 12 non-synesthetic control subjects. In controls, a network of regions was recruited during the observation of touch to a human relative to an object (including primary and secondary somatosensory cortex, premotor regions, and the superior temporal sulcus). Similar brain regions were also activated during actual touch, indicating that observing touch to another person activates a similar neural circuit as actual tactile experience—the mirror-touch system. “C” recruited a similar network of regions, but showed hyperactivity in many of these regions (including the primary and secondary somatosensory cortices). This hyper-activity was interpreted as the neural correlate of C's synesthesia, with the suggestion that C's overt experiences of touch when observing touch to others may be a function of hyper-excitability of normal somatosensory mirroring mechanisms (Blakemore et al., 2005).

While hyper-excitability of somatosensory mirroring mechanisms may be a correlate of mirror-touch synesthesia, precisely what contributes to mirror-touch synesthetes showing increased cortical excitability within the mirror-touch system when observing touch to others is somewhat elusive. It is not the case that the somatosensory system is hyper-excited in some global (i.e., context-free) sense. For instance, in a recent group fMRI study (Holle et al., under revision) there was evidence of hypo-excitability (in mirror-touch synesthetes relative to the control group) within somatosensory regions when observing touch to dummy faces. The latter stimuli do not tend to elicit synesthetic touch. As such the activity within the somatosensory network seems to be differently modulated (or gated) in synesthetes relative to controls.

In controls, behavioral evidence from an interference paradigm involving real touch (to one's own face) and the sight of touch (to an observed face) shows that visuo-tactile interference is greatest when self-other similarity is greater; for instance, in terms of visual appearance or even political opinions (e.g., Serino et al., 2008, 2009). One plausible suggestion is that faulty self-other monitoring mechanisms may lead to a disinhibition of normal somatosensory mirror mechanisms in individuals with mirror-touch synesthesia (Banissy et al., 2009; Fitzgibbon et al., 2012). In line with this, recent findings indicate that observing touch to others not only evokes overt tactile sensations in mirror-touch synesthetes, but also elicits changes in mental representations of the self (Maister et al., 2013). In that study the “enfacement illusion” was used to examine self-representations in developmental mirror-touch synesthesia. In the typical enfacement illusion participants are shown a series of images of morphed faces containing varying proportions of the participants face or an unfamiliar other, and are asked to indicate the extent to which the face looks like the self. They then view a video in which another person is being touched that is in synchrony and congruent with felt touch that is delivered to the participants face. This synchronous mapping between observed and felt touch leads participants to report an increase in perceived similarity between the other and themselves. That is to say that after experiencing synchrony between observed and felt touch, the images that participants had initially perceived as containing equal quantities of self and other became more likely to be recognized as the self (i.e., they show a self-other blurring where they begin to incorporate more of the other into representations of themselves—Tsakiris, 2008; Tajadura-Jimenez et al., 2012). For mirror-touch synesthetes, this self-other blurring was shown to occur in the absence of felt touch being applied to their own face, implying that simply viewing touch to others evokes a change in self-representations in mirror-touch synesthesia (Maister et al., 2013).

Potential candidate neural regions that may mediate a relationship between self-other processing and neural activity in the mirror-touch system include the inferior parietal lobule, temproparietal junction (TPJ), and anterior insula (see Northoff et al., 2011 for review of brain areas involved in representing and distinguish self from other). In the context of mirror-touch synesthesia, regions of particular note are the anterior insula and TPJ. In the functional neuroimaging study by Blakemore et al. (2005) the only brain region that was shown to distinguish between synesthete “C' and the control group was neural activity in the anterior insula. The anterior insula has been linked to self-other processing in several domains, including self-face recognition (e.g., Devue et al., 2007), body ownership (e.g., Tsakiris et al., 2007), and perspective taking (e.g., Ruby and Decety, 2001). It is also known to have structural connections with neural regions involved in the mirror-touch system, including the secondary somatosensory cortex (Mesulam and Mufson, 1985): in this context it is notable that although our recent neuroimaging study of a group of mirror-touch synesthetes did not observe functional differences in the anterior insula (Holle et al., under revision), we did see cortical excitability differences localized to the secondary somatosensory cortex, which may be mediated by functional connectivity with the anterior insula.

A further candidate that may contribute to atypical self-other processing in mirror-touch synesthesia is the TPJ. The TPJ is also commonly linked to self-other representations, including agency discrimination (e.g., Farrer and Frith, 2002), perspective taking (e.g., Aichhorn et al., 2006), empathy (e.g., Völlm et al., 2006), and the online control of representations between self and other (e.g., Santiesteban et al., 2012). Recent findings indicate that mirror-touch synesthetes show structural brain differences relative to controls within the right TPJ (namely, reduced gray matter volume; Holle et al., under revision), suggesting broader cortical difference in mirror-touch synesthesia beyond regions involved in vicarious somatosensory mirroring. This area may therefore also contribute to atypical self-other processing in mirror-touch synesthesia (e.g., Aimola-Davies and White, 2013; Maister et al., 2013), which in turn may modulate somatosensory mirroring in mirror-touch synesthesia.

In a broader context, it is also interesting to consider the extent to which differences in cortical mechanisms related to self-other processing may contribute to broader traits observed in developmental mirror-touch synesthesia. For example, we have previously reported that developmental mirror-touch synesthetes show heightened levels of emotional empathy relative to controls (Banissy and Ward, 2007), and it is fairly clear to see how a blurring between the self and other may be useful in facilitating this capacity. However, one may also ask whether there may be circumstances where atypical self-other monitoring may lead to less beneficial consequences. One prediction may be that developmental mirror-touch synesthetes will show reductions in capacities that are dependent on their ability to engage online control of the representations of the self or other (e.g., agency discrimination). This remains to be determined with future studies. What is clearer, however, is that it would seem unlikely that alterations in self-other processing would lead solely to mirror-touch synesthesia; rather one would expect that mirror-touch synesthesia may be one of a constellation of traits associated with atypical mechanisms of self-other representation.

In sum, individuals with mirror-touch synesthesia experience tactile sensations on their own body when simply observing touch to others. While the majority of explanations related to this condition have focused around hyper-active somatosensory mirroring, relatively less has focused on the important role that self-other processing may play in modulating somatosensory mirroring mechanisms. Despite this, there is growing evidence to suggest atypical self-other representations in mirror-touch synesthesia and further work is needed to determine the relationship between neural regions involved in self-other processing and the mirror-touch system, in both mirror-touch synesthesia and typical adults.

## Footnotes

^1^These two spatial frames of reference are consistent with neurophysiological findings in primates documenting anatomical and mirrored spatial frames of reference that mediate bimodal visual-tactile cells in the macaque parietal cortex. These cells respond when the monkey is touched and when the monkey observes touch to the same body part of someone else (Ishida et al., 2009).
